# Robotic sacrocolpopexy: a game worth playing? A critical literature analysis

**DOI:** 10.3389/fsurg.2025.1561976

**Published:** 2025-03-07

**Authors:** Hussein Mansour Jamaleddine, Nour Khalil, Rana Aoun, David Atallah

**Affiliations:** ^1^ Department of Gynecology, University of Saint Joseph Faculty of Medicine, Hotel Dieu de France Hospital, Beirut, Lebanon; ^2^ Department of Urology, University of Saint Joseph, Hotel Dieu de France Hospital, Beirut, Lebanon

**Keywords:** prolapse, robotic, sacrocolpopexy, mesh, laparoscopy

## Abstract

Robotic sacrocolpopexy is an advanced minimally invasive technique for the surgical management of urogenital prolapse. It offers superior precision, reduced blood loss, and lower conversion rates compared to traditional approaches. However, longer operative times, higher costs, and the need for specialized training remain the most significant challenges of robotic surgery. The advantages of robotic sacrocolpopexy are reduced intraoperative complications, lower blood loss, and decreased conversion rates compared to traditional approaches. However, it was described to involve longer operative times, increased costs, and the need for a specialized training. Additionally, the technique shows significant potential for reducing complications in obese patients and improving cosmetic outcomes. Comparative studies highlight that robotic and laparoscopic sacrocolpopexy yield similar long-term outcomes, with differences primarily in operative time and cost-efficiency robotics. The lack of standardized protocols remains a limitation, and long-term data on durability and cost-benefit analyses are needed. Future research should prioritize optimizing outcomes, reducing costs, and improving accessibility to robotic urogynecologic surgery.

## Introduction

Urogenital prolapse is a prevalent condition, with approximately one in eleven women requiring surgical intervention in their lifetime (1).

The management of prolapse has evolved significantly—from the early use of pessaries, including rudimentary devices such as grenadines (2), to more sophisticated surgical approaches like abdominal sacrocolpopexy, first described by Lane in 1957 (3).

For years, the vaginal route was considered the preferred approach for prolapse repair due to its minimally invasive nature. However, the emergence of minimally invasive surgery has significantly changed this perspective. Laparoscopic sacrocolpopexy is now considered the standard of care for the management of severe apical urogenital prolapse, in patients suitable for general anesthesia (4). More recently, robotic-assisted sacrocolpopexy (RSC) is gradually replacing laparoscopic sacrocolpopexy (LSC) in institutions with resources to adopt this technology.

Early robotic studies, dating back to 2004, introduced a hybrid approach where vesicovaginal dissection was performed laparoscopically, followed by robotic-assisted mesh placement and suturing (5). Nowadays, the entire procedure can be performed robotically (6).

Despite these technological advances, controversy persists regarding the cost-benefit balance of RSC. Advocates emphasize its superior dexterity, enhanced 3D visualization, and ergonomic advantages, which may facilitate faster learning curves for novice surgeons. However, critics argue that RSC is associated with longer operative times, higher costs, and the absence of tactile feedback, which is crucial for sacral promontory dissection.

This review aims to critically evaluate robotic sacrocolpopexy, comparing its clinical outcomes, safety profile, and cost-efficiency with laparoscopic and open techniques.

### 1 Cohort studies: feasibility

With the progressive deployment of robotic surgery in hospitals, the results of small, often single-centered series are progressively published. They describe the outcomes of patients who have undergone robotic sacrocolpopexy. It is evident that this surgical approach can be used, as short-term follow-up studies (up to 2 years) report low complication rates and satisfactory anatomical correction (7–11). The average operative time is approximately 3 h with however the main obstacle to this technique being the cost of acquiring a robot.

To date, long-term studies on patients who have undergone robotic sacrocolpopexy are still lacking. Regarding success rates, findings vary across studies, with rates reported at 75% at 22 months (12), 60% at 50 months (13), and 90% at 5 years (14) (based on a series of 250 patients).

A major challenge in evaluating robotic sacrocolpopexy (RSC) is the lack of standardized definitions for surgical success, recurrence, and complications, leading to inconsistencies across studies. Success is commonly assessed through objective anatomical criteria, such as POP-Q stage ≤1, but subjective patient-reported outcomes and quality-of-life measures also play a crucial role (12). Recurrence rates vary widely depending on whether studies define failure as any prolapse beyond the hymen (POP-Q ≥ 2) or only cases requiring surgical reintervention (13). Similarly, mesh erosion rates are inconsistently reported, with some studies including asymptomatic findings, while others focus only on symptomatic cases requiring excision (15). These variations hinder direct comparisons between RSC, laparoscopic sacrocolpopexy affecting the interpretation of long-term outcomes.

The surgical technique is detailed in several publications, with comprehensive descriptions of each operative step (16). Surgical videos are also available online. The technique varies slightly among surgeons, which is not unusual, as similar variations were already present in the laparoscopic technique. While the main principles remain consistent, there is no consensus on certain details, such as the design of the mesh, the method of mesh fixation or the number of sutures required.

### 2 Comparison with the vaginal approach

Before addressing this section, it is important to note that in 2019, the Food and Drug Administration (FDA) issued a mandate to withdraw the use of vaginal meshes for prolapse repair (17), specifying that the decision would be reviewed 36 months later. After this period, the decision was upheld, as the FDA determined, based on available evidence, that the benefits did not outweigh the risks associated with transvaginally placed meshes for prolapse repair. Additionally, the FDA has continued to monitor the safety of these meshes in women who had already undergone such procedures (18). Ever since, the use of prostheses for vaginal prolapse repair has no longer been recommended due to their high morbidity and the emergence of minimally invasive abdominal alternatives.

However, some retrospective studies have compared outcomes between robotic sacrocolpopexy and vaginal reconstruction with prostheses for genital prolapse (19, 20) (Table 1). The vaginal approach is significantly faster but associated with higher blood loss. There is no significant difference in hospitalization duration between the two approaches, but *de novo* stress urinary incontinence is more prevalent with the vaginal approach (20).

**Table 1:** Summary of the comparison of the main metrics between robotic, laparoscopic, open sacrocolpopexy and vaginal surgery.

Parameter	Robotic sacrocolpopexy	Laparoscopic sacrocolpopexy	Open sacrocolpopexy	Vaginal surgery
Operative time (minutes)	180–240 (42)	120–180 (36)	210–300 (22)	90–120 (20)
Blood loss (ml)	50–100 (35)	50–150 (35)	300–600 (22)	200–400 (20)
Recurrence rate (%)	5–15 (12–14)	5–12 (35)	10–18 (22)	20–30 (20)
Mesh Erosion rate (%)	3–5 (15)	3%–5% (15)	3–6 (15)	7–12 (18)
Hospital stay (days)	1–2 (22)	1–2 (22)	3–5 (22)	1–3 (20)
Cost per case (USD)	8,000–12,000 (74)	4,000–6,500 (74)	6,000–8,500 (74)	2,000–4,000 (78)
Conversion rate (%)	<2 (41)	2–5 (41)	N/A	N/A
Vascular injury	Rare, <1% (51)	Rare, <1% (51)	Higher risk (22)	Minimal risk (20)
Nerve injury	Possible (57)	Possible (57)	Higher risk (22)	Uncommon (20)
Postoperative pain	Mild-moderate (33)	Mild-moderate (33)	More common (22)	Variable (20)
Bowel injury (%)	0–8 (53)	0–5 (53)	1%–3% (22)	Rare (20)
Port-site herniA	Possible, but rare (55)	Possible (55)	N/A	N/A

The complication rate is reported to be similar between the groups; however, the sample sizes are too small to compare major complications (e.g., gastrointestinal, vascular, or urinary) associated with abdominal approaches.

### 3 Comparison with the open approach

The validation and acceptance of minimally invasive techniques across all surgical fields must first demonstrate superiority over the open approach. Indeed, minimally invasive surgery in general often entails higher costs, a steeper learning curve, and requires a well-trained medical team (21). This applies as well to pelvic organ prolapse surgery.

Comparisons between different approaches to sacrocolpopexy began with comparisons to the open approach (Table 1). Early reports comparing perioperative, hospitalization, and follow-up outcomes between robotic and open sacrocolpopexy showed a longer operative time for robotic surgery but reduced blood loss and identical POP-Q improvements at six weeks (22).

One of the major advantages of minimally invasive surgery is the ease of managing obese patients. Open surgery in obese patients presents significant technical and surgical challenges for the surgeon, potentially compromising the surgical outcomes and increasing the risk of blood loss and injury to adjacent organs. Since obesity is a risk factor for prolapse, this population is a key group of interest for sacrocolpopexy. Studies have shown that obese patients (BMI > 30) can undergo minimally invasive sacrocolpopexy, whether laparoscopic or robotic, without differences in outcomes between the two techniques (23).

Furthermore, a key advantage of minimally invasive approaches over open surgery lies in improved cosmetic outcomes, with small incisions instead of a large abdominal incision. However, this concept has been criticized. In fact, cosmetic appearance is not always a primary concern for patients undergoing prolapse surgery (24). Some studies indicate that functional outcomes, such as prolapse resolution and symptom relief, hold greater significance for patients than scar size (25). Additionally, certain patient groups, particularly older individuals or those with a history of multiple surgeries, may prioritize durability and long-term success over cosmetic benefits (26). This suggests that while smaller incisions offer an aesthetic advantage, their clinical relevance in patient decision-making remains debated.

An interesting comparison exists between the mini-laparotomy approach (a small horizontal abdominal incision of 5–6 cm located two fingers above the pubic symphysis) and minimally invasive approaches (laparoscopy and robotic surgery) (27). Operative time was significantly shorter in the mini-laparotomy group (less than half the time), although vaginal repairs were more frequently performed in this group. Patients were able to be discharged on postoperative day 0 or day 1, regardless of the approach, and no differences were observed in postoperative complication rates. This would be relevant for patients unable to tolerate general anesthesia due to comorbidities (often age-related), as the minimally invasive approach was associated with longer operative times.

### 4 Comparison with Laparoscopy

The results of comparisons between the open and robotic approaches somewhat reflect studies previously conducted between laparoscopy and the open approach (Table 1). The most significant challenge—and a critical discussion point in urogynecological circles—is determining whether robotics truly offers a technical advantage and improves postoperative outcomes in sacrocolpopexy.

The main issue with laparoscopic sacrocolpopexy lies in the technical challenges it demands, particularly due to the extent of dissection required in the deep pelvis and the need for suturing in difficult-to-access locations (28).

One study demonstrated that for novices, 15 h of suture workshops were equivalent to performing 30 cases of sacrocolpopexy in terms of reducing operative time (29). Furthermore, a learning curve of 60 cases (a significant number) was required to effectively minimize complications (29).

The major advantage of robotic surgery lies in its ease of dissection and suturing, facilitated by the angulation of the robotic arms (with its 7 degrees of freedom) (30). Additionally, robotics offers superior 3D visualization with magnification that is not always achievable in conventional laparoscopy. The fourth robotic arm and the camera's stability also mitigate the potential bias of an inexperienced assistant, which can sometimes render laparoscopic procedures cumbersome (30).

However, the tactile feedback, which is sometimes critical during sacrocolpopexy for locating the sacral promontory, is absent in robotic systems. Dissection of the sacral promontory is a key step that can lead to catastrophic outcomes if vascular injury occurs at this site. Moreover, the surgeon is not at the bedside during robotic procedures, leaving the assistant solely responsible for manipulating the vaginal retractor, whose position is essential for optimal anterior and posterior dissection planes. In contrast, during laparoscopic procedures, the surgeon can promptly assist in positioning the retractor if needed.

Another advantage of robotic surgery, though not extensively studied in clinical trials, is the shorter learning curve for young surgeons compared to other minimally invasive techniques (as the robotic technique is inherently simpler than conventional laparoscopy) (31).

The use of 3-dimensional vision in laparoscopic and robotic surgery enhances depth perception, precision, and learning efficiency. A meta-analysis by Restaino et al. (confirmed that 3D visualization improves surgical performance and accelerates skill acquisition, reinforcing the benefits of robotic platforms in training young surgeons (32). An interesting study comparing surgical parameters of patients operated on exclusively by surgeons vs. those where senior residents performed approximately 50% of the procedure showed no differences in surgical or postoperative outcomes (33). Notably, the residents in this study had undergone simulator training, wet-lab practice, and mastered the surgical technique through instructional videos and observation.

This highlights how robotics facilitates quicker acquisition of surgical skills by young operators.

Several studies and reviews, primarily retrospective, have compared laparoscopic and robotic approaches for sacrocolpopexy, yielding variable results. One study comparing 160 patients who underwent laparoscopic sacrocolpopexy to 54 patients who underwent robotic sacrocolpopexy found a higher recurrence rate of POP in the robotic group (34).

A systematic review of 2,115 patients conducted in 2020 revealed that robotic procedures were significantly longer, involved less blood loss, and had fewer intraoperative complications (and lower conversion rates) compared to laparoscopic procedures (35).

A randomized controlled trial involving 100 patients within the same institution compared robotic vs. laparoscopic sacrocolpopexy and found no significant differences between the two groups, except for a longer operative time in the robotic group, attributed to the inclusion of docking time in the calculation (36).

Conversely, another study demonstrated that robotic procedures were faster than laparoscopic procedures, with similar postoperative outcomes and complications (37).

Other studies comparing the two techniques for smaller groups found no differences in surgical or postoperative parameters (recurrences and complications) (38–40).

Regarding operative time, it is important to note that variations exist in studies on robotic surgery, as some authors exclude docking time from the total operative duration.

An interesting finding from a large American database was the lower conversion rate in the robotic group compared to the laparoscopic group (41). The most common reasons for conversion were extensive adhesions and injuries to difficult-to-identify organs. Since the number of obese patients was similar in both groups, it can be concluded that robotics facilitates surgery in obese patients.

It is worth noting the lack of standardization in studies. For example, some surgeons may be highly experienced laparoscopists or robotic surgeons, which can bias the quality of the procedure and postoperative follow-up (16). Rarely do studies mention the surgeons' experience. For instance, a randomized controlled trial strictly comparing operative times between laparoscopic and robotic procedures found laparoscopy to be faster, even for suturing (average 30 min faster) (42). This finding is atypical since robotics is generally expected to simplify and expedite such operative steps, potentially at the cost of longer docking times, but no longer dissection or suturing times.

Regarding postoperative pain management, including acetaminophen use and opioid requirements, no differences were found between the two minimally invasive approaches (43).

In this context, one advantage of minimally invasive surgery, whether laparoscopic or robotic, is the feasibility of outpatient surgery for these procedures. A feasibility study was conducted for procedures starting before noon, involving only anterior mesh placement and excluding patients requiring conversion (44). Among the 70 patients studied, no readmissions were necessary during the early postoperative period.

In conclusion, to date, the two minimally invasive techniques for sacrocolpopexy are considered comparable and should be chosen based on the surgeon's preference.

### 5 Technical Considerations

Initially, the robotic minimally invasive surgical technique was identical to the laparoscopic technique. Several questions arise regarding the best ways to optimize operative times and reduce short- and medium-term morbidity risks.

Currently, there is no clear consensus on the optimal number of sutures required (45) or their placement on the vagina to ensure the most effective fixation of the mesh (46). Historically, the sutures used for mesh fixation have been non-absorbable. However, one study explored the use of slowly absorbable sutures and demonstrated a lower erosion rate, while the recurrence rate remained the same (47). Drawing conclusions based on a single study is premature, but the idea is intriguing, particularly as the surgical population increasingly includes younger patients. Moreover, in the rare cases where chronic pain develops postoperatively, it may be related to the presence of permanent materials.

Traditionally, meshes have been pre-peritonealized, with the peritoneum opened during dissection and closed at the end of the procedure. A study compared a small cohort of patients where the peritoneum was not closed to another where it was (48), finding that omitting peritoneal closure significantly reduced postoperative pain, dyspareunia, and operative time without increasing postoperative complications. Similarly, another study prospectively followed a series of patients without peritonealization for an average of 19 months, searching for gastrointestinal complications but found none (49).

Conversely, there are case reports where the sacrocolpopexy mesh has been implicated in intestinal obstruction, even occurring very late postoperatively (50).

These findings should therefore be interpreted with caution.

### 6 Complications

The complications of robotic sacrocolpopexy are inherent to the technique itself, compounded by those associated with the surgeon's lack of experience. RSC's complications appear to be similar to those of conventional laparoscopic sacrocolpopexy (51).

a. Intraoperative complications

Intraoperative complications include injuries to adjacent organs. The most common injury would be to the bladder, then to the vagina, less frequently to the digestive system, and least but most feared to the vessels.

Bladder injuries are inconsistently reported, with an incidence of approximately 3% (52). If the injury is small and closure is watertight, the anterior mesh can be placed, with an extension of bladder catheterization. Vaginal injuries have also been reported, ranging from 0% to as high as 24% in one study (53). Vaginal injuries convert the surgical field into a contaminated site, generally precluding mesh placement.

The same study by Anand et al. reported high complication rates (10% cystotomy and 24% vaginal injury), attributing these to patients who had undergone previous hysterectomy.

More severe injuries involve the digestive tract, with incidence rates ranging from 0% (36, 54) to 8% (53). These often require the involvement of gastrointestinal surgeons to manage the treatment, depending on the affected organ.

The most feared complication is vascular injury, particularly to the middle sacral vessels during promontory dissection or, more rarely, to the iliac vessels. Though rare (<1% incidence), these injuries often necessitate conversion to an open approach.

b. Postoperative complications

Early postoperative complications include gastrointestinal issues such as ileus or bowel obstruction (10), which rarely require surgical intervention. Postoperative ileus is often linked to extensive dissection or prolonged pneumoperitoneum (10). It can be prevented by gentle adhesiolysis, avoiding excessive pneumoperitoneum and early postoperative feeding and mobilization.

Trocar site hernias are another concern, reported in up to 4% of cases, typically presenting around four days postoperatively, most commonly at the umbilical site but occasionally at lateral trocar sites (53). Most of these require surgical repair. Hernias have been reported at 8 mm trocar sites, which are generally considered unlikely to develop hernias. This may be due to excessive trocar manipulation during surgery (55).

Massive subcutaneous emphysema is another rare complication, often associated with prolonged operative times, multiple trocar sites, and significant pneumoperitoneum. This can lead to systemic hypercarbia and, in extreme cases, gas embolism. Three cases of major subcutaneous emphysema related to robotic sacrocolpopexy have been reported (56), all resolving within 5–7 days postoperatively without sequelae.

Rare but serious nerve injuries can occur due to prolonged Trendelenburg positioning combined with gynecological flexion (57). The most commonly reported injuries include femoral nerve neuropathy due to hip hyperflexion, brachial plexus injury from shoulder compression, and peroneal nerve damage from leg positioning in stirrups (57, 58). These can be mitigated through careful patient positioning, including adequate padding, limiting extreme hip flexion, and avoiding extensively long interventions (59).

c. Mortality rate

Regarding mortality, a meta-analysis of minimally invasive benign gynecological procedures reported a mortality rate of 1:1,246 for all analyzed cases of minimally invasive sacrocolpopexy (laparoscopic and robotic) (60). It is important to note that this surgery is often performed on elderly, frail, and comorbid patients.

d. Late complications

Late complications most frequently involve prosthesis exposure, with rates averaging 3%–5% at five years (15, 61). Two-thirds of these are asymptomatic and detected only on clinical examination (62). Rarely, exposure may occur per rectum (posterior mesh) (63).

Most important techniques to avoid mesh erosion include avoiding excess vaginal dissection, using microporous polypropylene mesh (47), and performing a regular post-operative follow-up to detect asymptomatic erosions.

Another rare but serious late complication is infective osteitis or spondylodiscitis at the anchoring site on the sacral promontory. Presenting as back pain, sometimes accompanied by fever and systemic symptoms, these infections require vigilance as they may progress to severe outcomes such as fistula formation (53) or fungal infections (64). They often constitute a diagnostic challenge.

e. Robot-specific complications

There is a category of complications specific to robotic surgery, related to the lack of tactile feedback, use of rigid and fixed robotic arms, and the surgeon's physical distance from the operative field (57). These complications have not been exclusively studied in robotic sacrocolpopexy patients. Preventing these issues requires proper operative positioning (to avoid skin injuries), a skilled surgical team, and adequate training for the operator, including simulation modules and effective mentorship (65), to avoid excessive Trendelenburg positioning and prolonged operative times.

Moreover, concerns related to system failures or instrument malfunction would necessitate backup plans including laparoscopic conversion protocols.

f. Barbed suture-related complications

Barbed sutures such as V-Loc or Stratafix, simplify running sutures by eliminating the need for an assistant to hold the suture during the procedure (66). These sutures are nearly always used in robotic sacrocolpopexy for anterior and posterior peritonealization of the mesh. Rarely, mechanical obstruction due to volvulus (resulting in intestinal ischemia requiring resection) has been reported due to the remaining strand of the barbed suture (67). Suggestions to avoid this complication include replacing barbed sutures with other absorbable sutures, covering the remaining strand with adhesive material to prevent contact with bowel loops (68), or cutting the final strand very short (69).

### 7 Associated procedures during robotic sacrocolpopexy

Robotic sacrocolpopexy allows for the combination of additional procedures, particularly in complex prolapse cases.

a. Suburethral slings

As with laparoscopy, suburethral slings can be placed simultaneously for cases of stress urinary incontinence (SUI). SUI can sometimes become unmasked following prolapse repair. It can occur before or after the robotic procedure without affecting failure rates, complications, or the need for additional treatment (70).

b. Paravaginal repairs

A cohort study assessed the feasibility of paravaginal repairs for lateral or mixed prolapse. In addition to classic sacrocolpopexy, suturing the arcus tendineus fascia pelvis (ATFP) to the pubocervical fascia yielded good midterm results without prolapse recurrence (71).

c. Ventral rectopexy

Finally, ventral rectopexy can be performed for associated rectal prolapse (72). It can be done either as a standalone procedure or concomitantly with sacrocolpopexy using robotics. A series of 321 patients, including 170 with predominant rectal prolapse, demonstrated that these techniques could be combined without increasing complication rates (73), provided the operator is experienced.

### 8 Cost of RSC

The major disadvantage of robotic surgery is financial. The adoption of robotic-assisted surgery presents a significant financial challenge for healthcare institutions, particularly those with budget constraints. The upfront cost of robotic platforms, ranges from $1.5 million to $2.5 million per unit, with an additional annual maintenance cost of approximately $100,000–$170,000 (74).

Robotic instruments have a limited lifespan, and the platform requires annual maintenance.

Beyond the robotic system itself, the institution incur additional costs including: disposable robotic instruments and accessories, specialized training programs for surgeons and the staff (75) and longer operative times (42, 57).

Due to these financial constraints, robotic platforms are concentrated in high-volume tertiary care centers, often excluding smaller hospitals and resource-limited settings (74). This raises concerns about equitable access to advanced surgical techniques, as many patients may not have the option for RSC depending on healthcare systems and policies.

Some cost-minimization studies suggest that while RSC is initially expensive, its potential for shorter hospital stays and reduced complications could make it cost-neutral in the long term (76). A 2,012 study published in the *Journal of Urology* found robotic surgery to be less costly than open surgery due to shorter hospital stays, although it is unclear whether the annual maintenance cost of the robot was factored in (76). This claim remains controversial, as hospital stays reductions have also been reduced with laparoscopy, without the added cost of robotic platforms (35).

A detailed cost-analysis study incorporating operative time, risk of conversion, complications, and hospital stay duration used models like “Robotic Existing” and “Robotic Purchase” (74). These analyses examined cost-saving strategies such as increasing annual procedure volume and reducing hospitalization duration. However, the analysis concluded that robotic surgery remains the most expensive, followed by laparoscopy and then open surgery. Interestingly, having the same operating team assist with robotic surgeries significantly reduced operative times, thereby lowering the costs of robotic interventions (75).

A large study based on an American database compared the costs of robotic vs. laparoscopic sacrocolpopexy, showing that robotic surgery was more expensive per case, but the risk of readmissions and complications was similar between the two approaches (77).

A study comparing the cost of vaginal prolapse repair using mesh to robotic sacrocolpopexy demonstrated significantly lower costs for the vaginal approach. However, as vaginal mesh is no longer recommended, this comparison is outdated (78). Other techniques that would be of interest concern the ultra-minimally invasive surgery which includes both minilaparoscopic (3 mm trocar) and percutaneous endoscopic surgery. They can address urogenital prolapse by bilateral sacrospinofixation. Several studies observed UMIS benefits in terms of shorter hospital stay, better aesthetic outcomes, less postoperative discomfort, and increased patient satisfaction compared to traditional laparoscopic or robotic surgery (79).

Moreover, though not formally studied, surgeon experience could reduce the costs of robotic surgery. Experienced surgeons might use fewer instruments than novices, have shorter operative durations, and face lower risks of complications and readmissions (16).

The true economic debate surrounding RSC lies in the gap between institutional investments and system-wide healthcare savings. From an institutional perspective, hospitals must ensure high surgical volumes to offset costs, while from a healthcare system perspective, policymakers must assess whether robotic procedures reduce downstream costs (repeat surgeries, complications, readmissions) (77).

From a patient's point of view, while the financial burden is high, potential benefits include lower long-term complication rate, possible better quality of life and outcomes particularly in patients at high risk of surgical failure (obese, previously operated) (23). Unfortunately, data on lifetime cost-effectiveness remain limited.

The economic viability of RSC depends on patient selection, surgical volume, and institutional efficiency. Future research must focus on comprehensive cost-benefit analyses and long-term patient-centered outcomes, guiding healthcare policy decisions on whether RSC should be prioritized over laparoscopy in routine clinical practice.

### 9 Challenges and future perspectives

a. Uterine preservation

A recent meta-analysis compared laparoscopic sacrocolpo(hystero)pexy with and without total/subtotal hysterectomy (80, 81). They concluded that associating the hysterectomy would lead to higher success rates especially for anterior compartment prolapse with no significant differences in complications, recurrence and reoperation rates.

b. Management of sacrocolpopexy complications

Robotic surgery is increasingly being used to manage complications of sacrocolpopexy, such as mesh erosion or vesicovaginal fistula repair (82). The major advantage in such cases lies in the precision of dissection afforded by 3D magnified vision and EndoWrist technology, which facilitates identification of fibrotic planes in a surgically challenging pelvis.

c. Management of recurrent prolapse

An increasing number of studies report case series on the robotic correction of recurrent prolapse following previous surgeries (24), while emphasizing that the approach must be individualized for each case (25).

d. Management of complex prolapse

The literature includes several case reports describing robotic interventions for complex or atypical prolapse cases, such as post-cystectomy genital prolapse with colpocleisis in a patient with continent urinary diversion (73). Due to the fragility of the vaginal mucosa, the authors opted for a repair using mesh fixed to the levator muscles and Cooper's ligaments instead of laparoscopic sacrocolpopexy.

e. Use of autologous tissues

In light of controversies surrounding mesh use in vaginal prolapse repair and the withdrawal of such devices for this indication, pelvi-perineology teams are questioning whether abdominally placed meshes might also eventually be abandoned. This has spurred investigations into the use of autologous tissues for prolapse management (83). One study reported the use of fascia lata autografts in 12 patients, with no recurrences at one year (26). Interestingly, most of these patients had previously experienced complications with synthetic mesh.

Other repairs done laparoscopically consists in suspending the vaginal wall to the uterosacral ligaments previously called “Shull repair’’. It was done laparoscopically (31, 84) and would be easily done robotically. Learning curve consist in twenty cases and the results are satisfactory in a multicentric analysis.

f. Single-port robotic surgery

Another area of research involves the emergence of single-port robotic surgery. A video article has already documented a single-port robotic sacrocolpopexy (85). Furthermore, a narrative review has summarized several small studies on single-port laparoscopic or robotic sacrocolpopexy, although it is clear that this technique remains in its infancy (58). A randomized controlled trial comparing single-port to multi-port procedures found no significant differences in outcomes, except for longer operative times in the single-port group (54). A study analyzing the learning curve for single-port sacrocolpopexy indicated that significant reductions in operative time were achieved after the first 10 cases (86). It is certain that larger multi-center trials are needed to determine long-term efficacy and safety as well as cost-effectiveness analyses.

g. V-NOTES sacrocolpopexy

V-NOTES (transvaginal natural orifice transluminal endoscopic surgery) single-port has also been used for sacrocolpopexy (87). Two cases of robotic V-NOTES sacrocolpopexy were reported within a series of eight other laparoscopic V-NOTES sacrocolpopexies. V-NOTES is gaining popularity in gynecological minimally invasive approaches due to its unique transvaginal access. However, for sacrocolpopexy specifically, it is debatable whether this transvaginal approach might increase the risk of intravaginal mesh erosion, given the intentional breach of the mucosal barrier.

## Discussion

The emergence of robotic-assisted sacrocolpopexy has caused considerable debate in urogynecology. This surgical approach, has demonstrated notable potential in terms of precision, reduced intraoperative complications, and shorter hospital stays. However, it is essential to critically examine its position relative to established methods, especially laparoscopic, and open approaches. While robotic platforms offer enhanced visualization, superior dexterity, and ergonomic advantages (88), concerns persist regarding longer operative times, higher costs, and the absence of tactile feedback, particularly during sacral promontory dissection (16).

Comparative studies suggest that robotic and laparoscopic sacrocolpopexy yield similar long-term anatomical outcomes, with some advantages in terms of reduced intraoperative blood loss and lower conversion rates for robotic (35) although operative times were shown to be longer, with reported median differences of 30–60 min in most studies (42). Despite this, shorter hospital stays and improved ergonomics are commonly cited benefits of robotic surgery (22).

Another advantage is the shorter learning curve for robotic surgery, which may facilitate training for junior surgeons (33). However, other studies emphasize that high surgical volume and experience remain crucial for optimizing outcomes, irrespective of the chosen technique (16).

Despite these advantages, some studies report no significant differences in recurrence rates, complication profiles, or long-term outcomes between the two approaches. This raises important considerations for surgical decision-making, particularly regarding cost-efficiency and resource allocation within healthcare systems.

Robotic sacrocolpopexy allows for mesh placement in a controlled, visualized environment, potentially mitigating the risks of mesh erosion and exposure (15). Moreover, the ability to concurrently address associated conditions, such as urinary incontinence via suburethral sling placement, highlights the procedural versatility of robotic surgery (70). However, studies underscore the need for long-term data to better understand the durability of outcomes and identify patient subgroups that may benefit most from this approach.

As robotic platforms evolve, novel approaches such as single-port surgery (86) and V-NOTES (87) have emerged as innovative alternatives. These methods aim to further reduce invasiveness and improve cosmetic outcomes, although their application in sacrocolpopexy remains in its early stages. The potential shift towards using autologous tissues instead of synthetic mesh is another area of growing interest, particularly given the ongoing debates surrounding mesh-related complications (26). As new techniques and materials are introduced, comparative studies are essential to determine their relative safety, efficacy, and cost-effectiveness.

Ultimately, the choice of surgical approach must be individualized, accounting for factors such as patient comorbidities, prior surgical history, and anatomical considerations. While robotic sacrocolpopexy offers several potential benefits, laparoscopy remains a cost-effective alternative, and open surgery still plays a role in select cases (74). There is no universally ideal surgical technique; instead, the optimal approach depends on the specific context, surgeon preference, patient comorbidities, and individual expectations.

## Conclusion

This review highlights the key aspects of robotic sacrocolpopexy. While technological advances in surgery have significantly transformed the field, prolapse repair remains a functional surgery where patient needs and preferences must take precedence. Future research should focus on long-term comparative effectiveness studies, cost-benefit analyses, and patient-reported outcomes to better guide clinical decision-making.
